# Ocean Acidification Affects the Cytoskeleton, Lysozymes, and Nitric Oxide of Hemocytes: A Possible Explanation for the Hampered Phagocytosis in Blood Clams, *Tegillarca granosa*

**DOI:** 10.3389/fphys.2018.00619

**Published:** 2018-05-23

**Authors:** Wenhao Su, Jiahuan Rong, Shanjie Zha, Maocang Yan, Jun Fang, Guangxu Liu

**Affiliations:** ^1^Agriculture-Environment-Biology Group, College of Animal Sciences, Zhejiang University, Hangzhou, China; ^2^Zhejiang Mariculture Research Institute, Wenzhou, China

**Keywords:** ocean acidification, phagocytosis, cytoskeleton, NO, lysozyme

## Abstract

An enormous amount of anthropogenic carbon dioxide (CO_2_) has been dissolved into the ocean, leading to a lower pH and changes in the chemical properties of seawater, which has been termed ocean acidification (OA). The impacts of *p*CO_2_-driven acidification on immunity have been revealed recently in various marine organisms. However, the mechanism causing the reduction in phagocytosis still remains unclear. Therefore, the impacts of *p*CO_2_-driven OA at present and near-future levels (pH values of 8.1, 7.8, and 7.4) on the rate of phagocytosis, the abundance of cytoskeleton components, the levels of nitric oxide (NO), and the concentration and activity of lysozymes (LZM) of hemocytes were investigated in a commercial bivalve species, the blood clam (*Tegillarca granosa*). In addition, the effects of OA on the expression of genes regulating actin skeleton and nitric oxide synthesis 2 (*NOS2*) were also analyzed. The results obtained showed that the phagocytic rate, cytoskeleton component abundance, concentration and activity of LZM of hemocytes were all significantly reduced after a 2-week exposure to the future OA scenario of a pH of 7.4. On the contrary, a remarkable increase in the concentration of NO compared to that of the control was detected in clams exposed to OA. Furthermore, the expression of genes regulating the actin cytoskeleton and NOS were significantly up-regulated after OA exposure. Though the mechanism causing phagocytosis seemed to be complicated based on the results obtained in the present study and those reported previously, our results suggested that OA may reduce the phagocytosis of hemocytes by (1) decreasing the abundance of cytoskeleton components and therefore hampering the cytoskeleton-mediated process of engulfment, (2) reducing the concentration and activity of LZM and therefore constraining the degradation of the engulfed pathogen through an oxygen-independent pathway, and (3) inducing the production of NO, which may negatively regulate immune responses.

## Introduction

Ocean acidification (OA) has become a global environmental issue under the spotlight for the last decade (Kelly et al., 2011). Tremendous anthropogenic CO_2_ has been emitted since the Industrial Revolution, which has resulted in the rise of atmospheric CO_2_ levels from 280 to 390 μatm (Orr et al., 2005). Approximately 1/4 to 1/3 of emitted CO_2_ was absorbed by the ocean, which increases the concentration of H^+^ and alters the equilibrium of the oceanic carbonate system in surface seawater (Caldeira and Wickett, 2003; Sabine et al., 2004; Raven et al., 2005). The pH of surface seawater has decreased by 0.1 units compared to the pre-industrial level, and according to the prediction of the Intergovernmental Panel on Climate Change (IPCC), it may decrease to 7.8 and 7.4 by the end of the 21st century and the year 2300, respectively (Caldeira and Wickett, 2003). OA may therefore exert significant impacts on various aspects of organisms inhabiting the sea, such as growth (Wu et al., 2014), metabolism (Lannig et al., 2010), immune response (Liu et al., 2016), and sensory ability (Ashur et al., 2017), which on a large scale may pose a great threat to the entire marine ecosystem (Zhang et al., 2012).

The phylum Mollusca is one of the largest and most important groups in the animal kingdom, with approximately 130,000 living species (Parkhaev, 2017). As a category of invertebrates, mollusks mainly rely on non-specific immunity against pathogen challenges (Song et al., 2010). Nevertheless, recent studies have shown that OA could hamper the immune responses of various marine bivalve species, such as *Crassostrea gigas* (Wang et al., 2016), *Mercenaria mercenaria* (Ivanina et al., 2016), and *Mytilus edulis* (Mackenzie et al., 2014). Among all the parameters evaluating immune responses, the phagocytosis of hemocytes, one of the most important defense measures in non-specific immunity (Wang et al., 2012), including the engulfment and degradation of pathogen particles, has been widely used in previous studies (Liu et al., 2016; Shi et al., 2017; Su et al., 2017). Though an OA-induced reduction in phagocytosis has been reported in bivalve species, the mechanism manifesting this impact still remains unclear (Canesi et al., 2002; Sokolnikova et al., 2015).

Phagocytosis includes a series of sequential processes, such as the attachment of hemocytes to the targeted particles, the recognition of pathogen particles by pattern recognition receptors (PRRs), the engulfment facilitated by the cytoskeleton, and the degradation of foreign particles through oxygen-dependent and oxygen-independent pathways (Song et al., 2010). It has been suggested that OA could hamper the recognition of pathogen particles by hemocytes of marine invertebrates by affecting PRRs (Liu et al., 2016; Castillo et al., 2017). In addition, previous studies have shown that OA may lead to an increase in the production of reactive oxygen species (ROS), which is crucial for the oxygen-dependent degradation of engulfed pathogen particles (Duprécrochet et al., 2013; Wang et al., 2016). All of these previous studies contribute significantly to a better understanding of the potential mechanism underlying the OA-induced reduction in phagocytosis. However, the present knowledge of the impacts of OA on the processes of cytoskeleton-mediated engulfment and lysozyme-facilitated oxygen-independent degradation still remains scarce.

The engulfment of pathogen particles is facilitated by the ROS-sensitive cytoskeleton, mainly via the actin–myosin contractile system (Allen and Aderem, 1995). Therefore, theoretically, an increase in OA-induced ROS may exert a significant impact on the engulfment process by affecting the cytoskeleton of hemocytes (Tomanek et al., 2011; Goncalves et al., 2017). After engulfment, the phagosomes and lysosomes fuse together, and the engulfed target is destroyed within phagosomes mainly by lysozymes (the oxygen-independent degradation) or ROS (the oxygen-dependent degradation) (Buggé et al., 2007). However, since opposite results were reported in different species, the impact of OA on the lysozyme concentration still remains controversial (Munari, 2014; Bresolin et al., 2016). In addition, nitric oxide (NO) plays versatile roles in immune responses such as phagocytosis in which NO not only functions as an active antimicrobial molecule *in vivo* but also has been recognized as one of the most important immune regulators (Bogdan, 2001). Nevertheless, little is known about the effects of OA on the production of NO in hemocytes of marine bivalves to date.

The blood clam, *Tegillarca granosa*, is a traditional commercial marine bivalve species that is naturally distributed from the coasts of South Africa to the Asia Pacific (Shao et al., 2009; Peng et al., 2015). The blood clam occupies an important ecological niche in the intertidal zone and is therefore crucial for the material and energy flows of intertidal ecosystems (Liu et al., 2014). In addition, inhabiting in the intertidal zone where surface runoff converges into the sea, the blood clam is often challenged by various pollutants and deemed as an ideal marine bivalve species with which to investigate the impacts of environmental stresses on immune responses (Zhu et al., 2011; Liu et al., 2016; Shi et al., 2017; Su et al., 2017).

Therefore, in order to obtain a better understanding of the impact and mechanism of the effect of OA on the process of phagocytosis, the rate of phagocytosis, the abundance of the cytoskeleton component, the levels of NO, and the concentration and activity of lysozymes (LZM) of hemocytes under present and future OA scenarios were investigated in the blood clam, *T. granosa*. In addition, the impacts of OA on the expression of key genes regulating the actin skeleton and nitric oxide synthesis 2 (*NOS2*) were also analyzed in the present study.

## Materials and Methods

### Experimental Animals and Acclimation

Adult individuals of *T. granosa* with a shell length of 28.53 ± 0.52 mm were collected from Qingjiang, Wenzhou, and Zhejiang Provinces of China (28°28′ N and 121°11′ E) in July 2017. Before the commencement of the experiment, individuals were acclimated for a week in a 1000-L tank filled with 800 L of sand-filtered 24-h-aerated seawater (temperature at 22.18 ± 1.30°C, pH at 8.11 ± 0.02, and salinity at 20.67 ± 0.09). The seawater in the tank was replaced once daily 2 h after the individuals were fed with the microalgae *Platymonas subcordiformis* (Han et al., 2016).

### Ocean Acidification Stimulation and Seawater Chemistry Analysis

According to the near-future OA scenarios predicted by the IPCC, pH levels of 8.1, 7.8, and 7.4 were employed to simulate the pH levels at present and in the years 2100 and 2300, respectively. According to the method of Zhao et al. (2017a), the stimulation of the acidified scenario was achieved by bubbling dry air or a mixture of carbon dioxide and dry air with different but constant percentages.

After 1 week of acclimation, blood clams with similar sizes were selected out from the acclimating tank and allocated into separated experimental tanks filled with 30 L of seawater at desired experimental pH. Thirty individuals were randomly allocated to each of the three pH levels, and three replicates were performed for each pH tested. During the 2-week exposure, blood clams were fed with the microalgae *P. subcordiformis* 2 h before the replacement of seawater everyday. The seawater of each trial was replaced with freshly sand-filtered seawater or pre-acidified seawater once daily.

To ensure consistent seawater conditions throughout the experimental progression in each trial, pH_NBS_, salinity and temperature were monitored daily, and the total alkalinity (TA) was monitored once a week (**Table 1**). The pH_NBS_ of each level was measured by a pH meter (PB-10, Sartorius) calibrated with NBS standard buffers. Salinity was determined using a conductivity meter (Multi 3410 WTW, Germany). The water temperature was measured with a mercury thermometer. TA was obtained by a potentio-metric titration (Anderson and Robinson, 1946). The carbonate system parameters were calculated from the measured pH_NBS_, salinity, temperature, and TA values using the open-source program CO2SYS (Pierrot et al., 2006), with the constants supplied by Mehrbach et al. (1973) and refitted by Dickson and Millero (1987), along with the K_2_SO_4_ dissociation constant from Dixson et al. (2010).

**Table 1:** The seawater parameters during the 2-week incubation of *T. granosa* (mean ± SE).

Target pH	T (°C)	Sal (‰)	pH_NBS_	TA (μmol/kg)	*p*CO_2_ (μatm)	DIC (μmol/kg)	Ωara	Ωcal
pH 8.1 (control)	23.9 ± 0.2	20.68 ± 0.07	8.13 ± 0.01	2061.98 ± 6.47	496.38 ± 16.79	1933.05 ± 7.37	1.75 ± 0.05	2.85 ± 0.09
pH 7.8	23.7 ± 0.3	20.66 ± 0.05	7.80 ± 0.01	2066.41 ± 8.50.	1129.29 ± 26.14	2032.26 ± 9.17	0.88 ± 0.02	1.43 ± 0.03
pH 7.4	24.0 ± 0.1	20.64 ± 0.04	7.41 ± 0.01	2071.22 ± 6.59	2906.98 ± 28.11	2141.85 ± 6.85	0.37 ± 0.01	0.60 ± 0.01

*Partial pressure of CO_*2*_, dissolved inorganic carbon, and saturation state of aragonite and calcite were calculated from measured pH_*NBS*_, salinity, temperature, and TA values using the open-source program CO2SYS. T, temperature; Sal, salinity, TA, total alkalinity; *p*CO_*2*_, CO_*2*_ partial pressure; DIC, dissolved inorganic carbon; Ωara, aragonite saturation state; Ωcal, calcite saturation state.*

### Phagocytosis Assays

Following the methods described by Evelyne et al. (1991) and Shi et al. (2017), after a 2-week experimental treatment, three individuals were randomly picked from each trial for the phagocytosis assays. A yeast (Instant dry yeast, AngelYeast) suspension containing (1.45 ± 0.05) × 10^8^ yeast cells per ml was prepared by dissolving 7 mg of yeast powder in 1000 μl of Alsever’s solution (ALS, Noble Ryder) and supersonic vibration was used for a sufficient homogenized mixing. A volume of about 150 μl of hemolymph was extracted from each individual followed by a quick determination of hemocyte concentration with 20 μl hemolymph using a Neubauer’s hemocytometer (XB-K-25, Anxin Optical Instrument) under Nikon eclipse E600 microscopy at the magnification of 400×. Meantime, 100 μl of the hemolymph extracted was transferred into a 1.5-ml centrifuge tube, which was pre-filled with 100 μl of Alsever’s solution. After spinning at 1000 rpm for 15 s, 100 μl of supernatant was removed and a calculated volume of yeast suspension was added at the yeast-hemocyte rate of 10:1 (Cima et al., 2000). The yeast-hemocyte mixture was incubated at room temperature (25°C) for 30 min and then fixed with 100 μl of 2.5% glutaraldehyde. Blood smears were subsequently prepared and stained with Wright-Giemsa stain (G1020, Solarbio). The phagocytic rate for each individual was estimated using the blood smear under a Nikon eclipse E600 microscope at the magnification of 400×.

### Fluorescence Staining of the Cytoskeleton of Hemocytes

After a 2-week exposure to acidified seawater, three individuals from each pH level were selected for fluorescence staining of F-actin using Rhodamine Phalloidin (*amanita phalloides*, Cytoskeleton, Inc., #PHDR1) following the provided protocol and the method described by Kryukova et al. (2015). Briefly, the hemocytes were obtained as described above and then washed with PBS, followed by fixation with glutaraldehyde for 10 min at room temperature. After another 30-s PBS wash, the hemocytes were incubated in 0.1% Triton X-100 for 5 min. The blood cells were then washed again for 30 s with PBS and stained with 100 nM Rhodamine Phalloidin for 30 min in darkness. After three PBS washes, the hemocytes were collected and used to make slides for fluorescent analysis. Following the method described by Catapane et al. (2016) and Guo et al. (2017), the fluorescence intensity was determined under a Nikon Eclipse E600 microscope (at excitation and emission wavelengths of 535 and 585 nm, respectively) and then was used as an indicator for the abundance of cytoskeleton components. In brief, the fluorescence intensities of images captured were calculated and analyzed in Image Pro-Plus (IPP), using the equation: F = IOD/area, where F is the fluorescence intensity of the F-actin stained, integrated optical density (IOD) represents the total optical density value of the stained hemocyte area, and ‘area’ is the stained area of the hemocyte.

### Concentrations of NO in Hemocytes

Following the method described by Wang et al. (2008) and Shi et al. (2012), after a 2-week exposure to acidified seawater, three individuals from each pH level were selected for the concentration of NO in hemocytes. A volume of about 150 μl of hemolymph was extracted from each individual followed by a quick determination of hemocyte concentration with 20 μl hemolymph using a Neubauer’s hemocytometer (XB-K-25, Anxin Optical Instrument) under Nikon eclipse E600 microscopy at the magnification of 400×. The concentration of NO in hemocytes was determined by the nitrate reductase method using the NO assay kit A012 (Nanjing Jiancheng Bioengineering Institute). The NO content was analyzed after converting all ${\mathrm{NO}}_{3}^{–}$ in the testing sample into ${\mathrm{NO}}_{2}^{–}$ using nitrate reductase according to the manufacturer’s instructions. In brief, hemocyte sample of 100 μl was mixed with reagents 1 and 2 and then incubated at 37°C for 1 h. After adding reagents 3 and 4, the mixture was incubated for another 10 min, followed by spinning at 4000 rpm for 10 min. The chromogenic reagent was subsequently added to the collected supernatant. After 10 min of incubation, the absorbance values were determined at the wavelength of 550 nm using a microplate reader (Multiskan GO, Thermo). The concentration of NO were subsequently determined by putting the obtained absorption values into the standard curves and divided by the total hemocytes counts of the testing sample.

### Concentration and Activity of LZM in Hemocytes

The enzymatic activity and concentration of lysozymes of hemocytes were determined with ELISA kits FK-97441 and FK-97442 (FKBIO, Shanghai), respectively. As described above, after extraction and a quick determination of hemocyte concentration, 10 μl of the testing sample was mixed with 40 μl of diluent in a microwell plate and then incubated at 37°C for 30 min following the protocol provided. After the microwells were washed five times with a wash buffer, 50 μl of the conjugate reagent was added and then incubated at 37°C for another 30 min. The microwell was washed with the wash buffer again before adding 50 μl of each chromogenic reagent A and B. After 15 min of mixing in the dark, the stop buffer was used to terminate the chromogenic reaction. The values of absorbance were then determined at the wavelength of 450 nm using a microplate reader (Multiskan GO, Thermo). The concentration and activity of LZM of each sample were subsequently determined by referring to corresponding standard curves and calibrated with the hemocytes count.

### Gene Expression Related to Cytoskeleton Regulation and *NOS2*

After exposure to the corresponding experimental seawater, three individuals were randomly picked from each pH level for the gene expression analysis. Total RNA was extracted from 100 μl hemolymph using an EASYspin Plus tissue/cell rapid RNA exaction kit (Aidlab, RN2802) following the method described by Peng et al. (2016). The concentration and quality of the RNA were verified with a NanoDrop 1000 UV/visible spectrophotometer (Thermo Scientific) and gel electrophoresis, respectively. One microgram of high-quality RNA was reversely transcribed into first strand cDNA using the PrimeScript^TM^ RT reagent kit (TaKaRa, RR037Q).

Genes of actin-related protein 2 (*ARPC2*), actin-related protein 3 (*ARPC3*), Rho-associated protein kinase (*ROCK*), p21-activated kinase 2 (*PAK2*), Ras homolog gene family member A (*Rho*), GTPase Kras (*KRAS*), GTPase Mras (*MRAS*), and Ras-related C3 botulinum toxin substrate 1 (*Rac1*) out of the actin cytoskeleton regulation pathway and *NOS2* were analyzed in the present study. Primers and accession numbers for these tested genes and the reference 18S rRNA are listed in **Table 2**. All primers were synthesized by Sangon Biotech (Shanghai, China).

**Table 2:** Primers sequences for the genes investigated and the internal reference 18S rRNA (F and R after the dash line in the primer name indicate forward and reverse primer, respectively).

Primers	Sequence (5′ to 3′)	Accession no.
*18S*-F	CTTTCAAATGTCTGCCCTATCAACT	JN974506.1
*18S*-R	TCCCGTATTGTTATTTTTCGTCACT	
*ARPC2-*F	AGACGACGATGATATTGTGA	MG575744
*ARPC2-*R	GCATCTGTATTCTGTAGTTCC	
*ARPC3-*F	GCGAACAGGTCTGATGAA	MG575745
*ARPC3-*R	ACATAACCACCACTTGCTT	
*ROCK-*F	GGTGGTGATCTAGTCAATCT	MG575752
*ROCK-*R	GTCTGGCTTAACATCTCTATG	
*PAK2-*F	TTGTTACGATAGGAGATCCA	MG575749
*PAK2-*R	CCTGTTGCCACCTCTATT	
*Rho-*F	AGGTGGAGTTAGCATTATGG	MG575751
*Rho-*R	CTAAACTGTCGGGACTATCTA	
*MRAS*-F	TTGGTGGTGGTTGGTGAT	MG575747
*MRAS*-R	CCATCTATCTCCGTATGTTGTA	
*KRAS*-F	AGGTTGTTATTGACGGAGAG	MG575746
*KRAS*-R	AATCCTTCACCAGTTCTCAT	
*Rac*-F	ATGATGGTAGACAGTGTTCC	MG575750
*Rac*-R	ACGATGAAGGACTCACAAC	
*NOS2-*F	AGTGGCTGGTATATGTCAAC	MG575748
*NOS2-*R	CGCTTTCTGGAAACTATGTAG	

### Statistical Analysis

One-way ANOVAs followed by Tukey’s *post hoc* tests were conducted to detect differences in the rates of phagocytosis, the abundances of the cytoskeleton components, the levels of NO, and the concentrations and activities of lysozymes (LZM) of the hemocytes among treatments. For all analyses, the assumptions of normality and homogeneity of variances were assessed using Shapiro–Wilk’s and Levene’s tests, respectively. For cases where these assumptions were not satisfied by the raw data, such as the percentage data for the phagocytosis assays, the data were arcsine square root transformed prior to the analysis, following the method described by Su et al. (2017). Expression levels of each tested gene were compared with the control by a *t*-test. All of the statistical analyses were conducted using OriginPro 8.0, and a *p*-value of less than 0.05 was accepted as a statistically significant difference.

## Results

### The Impact of OA Exposure on Phagocytosis

**FIGURE 1:**
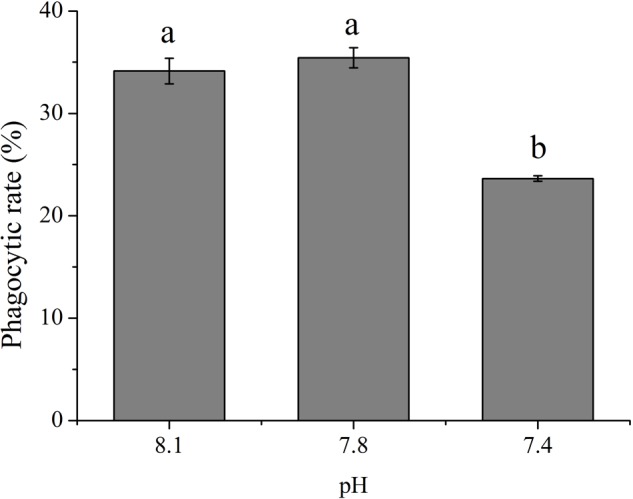
The rate of phagocytosis of *T. granosa* after 2-week exposure to present (pH 8.1) and predicted *p*CO_2_-driven ocean acidification (OA) scenarios (pH 7.8 and pH 7.4). All data was presented as means ± SE (*n* = 3) and different superscripts indicated a significant difference at *p* < 0.05.

As shown in **Figure 1**, OA exposure exerted significant impacts on the rate of phagocytosis of hemocytes. Though no significant difference was detected between the pH 7.8 group and the control, the phagocytic rate significantly (*p* < 0.05) decreased to only approximately 69.24% of that of the control when the clams were exposed to seawater acidified at a pH of 7.4.

### The Impact of OA on the Abundance of Cytoskeleton Components

**FIGURE 2:**
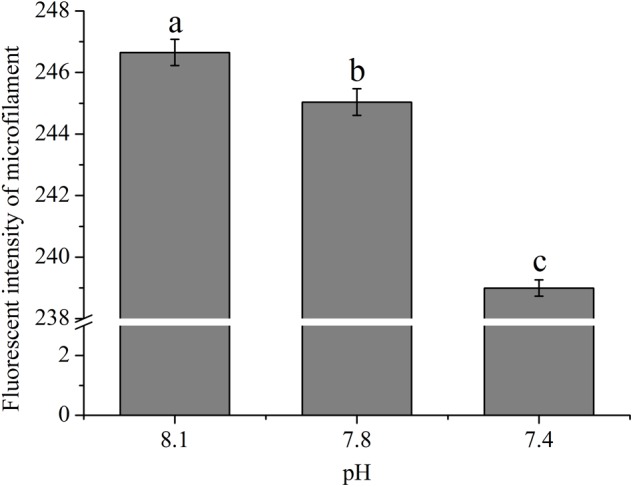
The fluorescence intensity of cytoskeleton (F-actin) of hemocytes of *T. granosa* after 2-week exposure to present (pH 8.1) and predicted *p*CO_2_-driven OA scenarios (pH 7.8 and pH 7.4). All data was presented as means ± SE (*n* = 4) and different superscripts indicated a significant difference at *p* < 0.05.

As shown in **Figure 2**, the abundances of cytoskeletons of hemocytes in terms of the fluorescence intensity of microfilament were significantly (*p* < 0.05) reduced when the clams were exposed to acidified seawater and were approximately 99.35 and 96.90% of that of the control for the pH 7.8 and pH 7.4 experimental groups, respectively.

### The Impact of OA on the Concentration of NO

**FIGURE 3:**
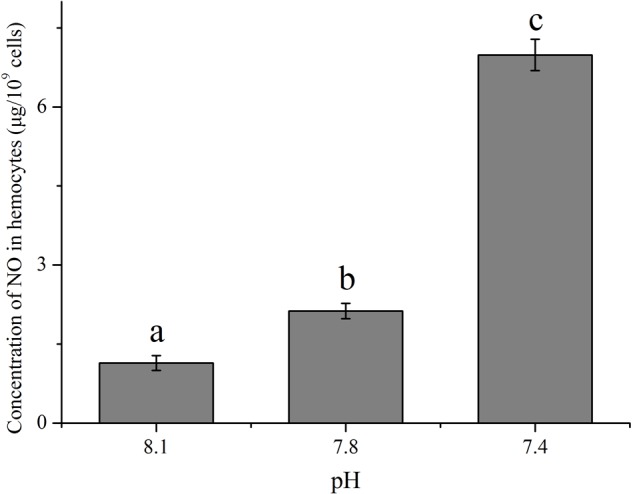
The concentration of nitric oxide (NO) in hemocytes of *T. granosa* after 2-week exposure to present (pH 8.1) and predicted *p*CO_2_-driven OA scenarios (pH 7.8 and pH 7.4). All data was presented as means ± SE (*n* = 3) and different superscripts indicated a significant difference at *p* < 0.05.

With an increase in the *p*CO_2_ level of the exposure seawater, an evident increase (*p* < 0.05) in the concentration of NO was detected in hemocytes of clams (**Figure 3**). After exposure to future OA scenarios for 2 weeks, the NO concentration in hemocytes increased to approximately 1.86 and 6.13 times that of the control for the pH 7.8 and pH 7.4 groups, respectively.

### The Impact of OA on the Concentration and Enzymatic Activity of LZM

**FIGURE 4:**
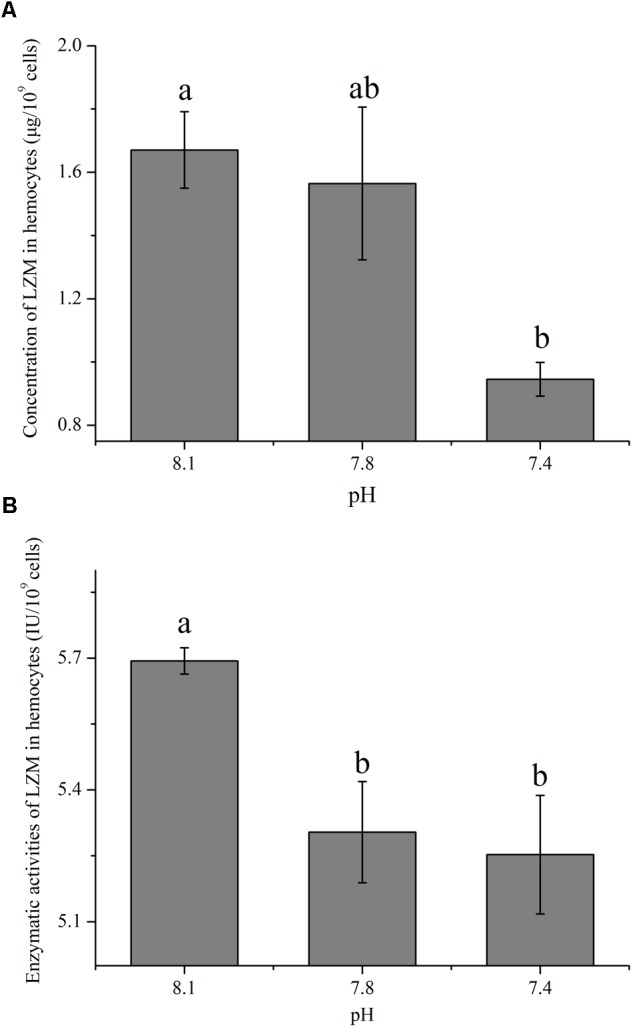
The concentration **(A)** and activity **(B)** of lysozyme (LZM) in hemocytes of *T. granosa* after 2-week exposure to present (pH 8.1) and predicted *p*CO_2_-driven OA scenarios (pH 7.8 and pH 7.4). All data was presented as means ± SE (*n* = 3) and different superscripts indicated a significant difference at *p* < 0.05.

The concentration and enzymatic activity of LZM in hemocytes under the present and near-future OA scenarios are shown in **Figures 4A,B**, respectively. OA exposure showed significant impacts on the concentration of LZM of hemocytes. Though no significant difference was detected between the pH 7.8 group and the control, the concentration of LZM significantly (*p* < 0.05) decreased to only approximately 56.60% of that of the control when the clams were exposed to acidified seawater at a pH of 7.4 (**Figure 4A**). As illustrated in **Figure 4B**, the enzymatic activities of LZM in hemocytes were significantly (*p* < 0.05) reduced by about 10% when the clams were exposed to acidified seawater.

### The Effect of OA Exposure on the Expression of Tested Genes

**FIGURE 5:**
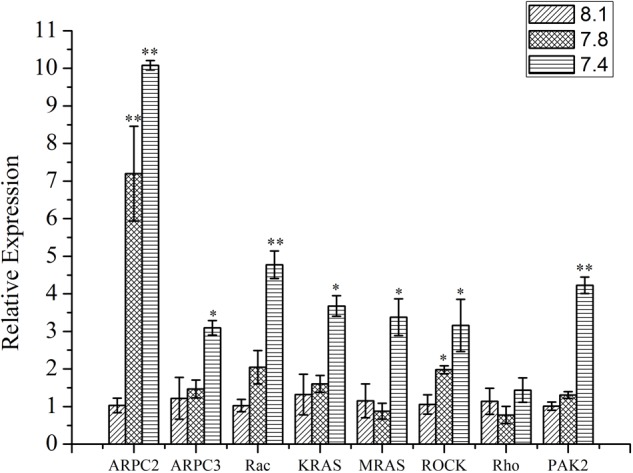
The expressions of key genes in actin cytoskeleton regulation pathway of *T. granosa* after 2-week exposure to present (pH 8.1) and predicted *p*CO_2_-driven OA scenarios (pH 7.8 and pH 7.4). All data was presented as means ± SE (*n* = 3). ^∗^*p* < 0.05 and ^∗∗^*p* < 0.01.

**FIGURE 6:**
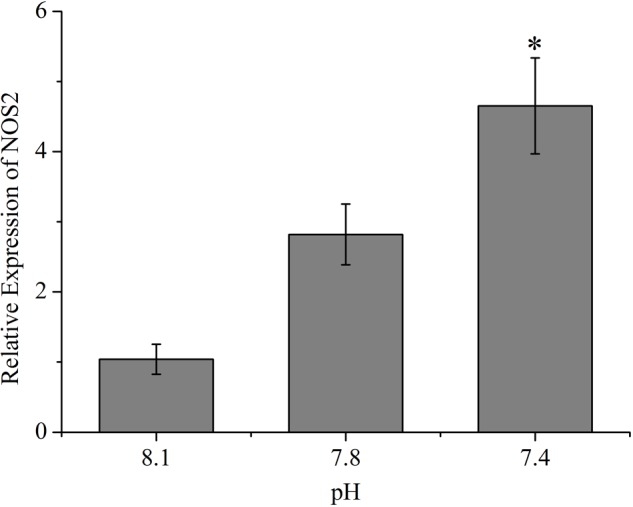
The expressions of nitric oxide synthesis 2 (NOS2) of *T. granosa* after 2-week exposure to present (pH 8.1) and predicted *p*CO_2_-driven OA scenarios (pH 7.8 and pH 7.4). All data was presented as means ± SE (*n* = 3). ^∗^*p* < 0.05.

As shown in **Figure 5**, except Rho, the expression of all the other tested genes (*ARPC2, ARPC3, Rac, KRAS, MRAS, ROCK*, and *PAK2*) from the actin cytoskeleton regulation pathway was significantly up-regulated (*p* < 0.05) after exposure of clams to acidified seawater. Similarly, the expression of the *NOS2* gene was significantly (*p* < 0.05) up-regulated after treatment of clams with acidified seawater, which were approximately 2.71 and 4.47 times that of the control for the pH 7.8 and pH 7.4 experimental groups, respectively (**Figure 6**).

## Discussion

**FIGURE 7:**
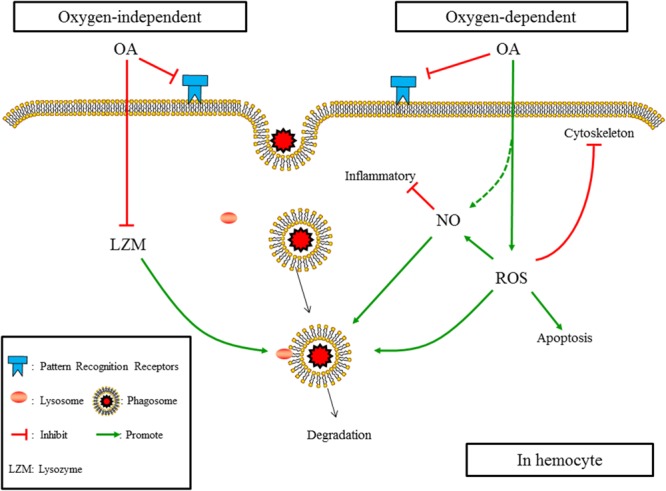
The summarized affecting mechanism of OA induced reduction in phagocytosis according to results obtained in the present study and those published previously. (1) OA hampers PRRs; (2) OA induces ROS, which increases apotosis of hemocytes, hampers cytoskeleton mediated engulfment, and alters oxygen-dependent degradation; (3) OA induces the production of NO, which may directly enhance the oxygen-dependent degradation but negatively regulates immune responses; (4) OA reduces the concentration and activity of lysozyme, which hampers the oxygen-independent degradation of engulfed particles.

When putting the results obtained in the present study and those reported previously together, as illustrated in **Figure 7**, the mechanism affecting the underlying OA-induced reduction in phagocytosis of hemocytes can be summarized as (1) OA hampers PRRs, which constraints the ability of hemocytes to recognizing foreign particles; (2) OA induces ROS, which increases apoptosis of hemocytes, hampers cytoskeleton mediated engulfment, and alters oxygen-dependent degradation; (3) OA induces the production of NO, which may directly enhance the oxygen-dependent degradation but negatively regulate immune responses; (4) OA reduces the concentration and activity of lysozyme, which hampers the oxygen-independent degradation of engulfed particles.

Previous studies have consistently shown that OA could induce the production of ROS in bivalve species, such as *C. gigas* (Wang et al., 2016), *M. coruscus* (Huang et al., 2016; Wu et al., 2016), and *C. virginica* (Tomanek et al., 2011). Though induced ROS suggests an enhanced oxygen-dependent degradation potency of hemoytes, it may cause apotosis of hemocytes and constrain the cytoskeleton-mediated process of engulfment. Three major cell types of hemocytes were identified in *T. granosa* (Zhu et al., 2011), among which the red granulocyte was predicted to have the highest ability of phagocytosis. According to previous results reported by Liu et al. (2016) and Su et al. (2017), OA could reduce the proportion and number of red granulocytes in blood clams probably due to OA-induced production of ROS. Therefore, the reduction of phagocytosis detected could be partially explained by the OA-induced production of ROS and subsequent apoptosis of hemocytes, especially those of red granulocytes.

F-actin, one of the essential components of microfilaments, which plays an essential role in the process of phagocytosis, is subject to the increase in ROS (Pollard and Cooper, 2009; Tomanek et al., 2011). Therefore, the reduction in the abundance of the cytoskeleton components in terms of the F-actin-specific fluorescence intensity detected in the present study could be attributed to the OA-induced production of ROS as well. Since the cytoskeleton-mediated process of engulfment is crucial for the phagocytic activity of hemocytes (Pollard and Cooper, 2009), OA could therefore hamper the engulfment of pathogen particles by reducing the abundance of the cytoskeleton component. Unlike the abundance of the cytoskeleton component, the key genes of the actin cytoskeleton regulation pathway were found to be up-regulated after OA exposure. Since *ARPC2* and *ARPC3* function as a complex to assemble the monomers into polymers in the process of producing F-actin from G-actin (Insall et al., 2001), the up-regulation of these two genes indicated an increased ability to produce the cytoskeleton component, which was also supported by the up-regulation of their upstream genes (*MRAS, KRAS*, and *Rac*). In addition, since *PAK2* stabilizes actin (Bokoch, 2003), the up-regulation of *PAK2* indicated an increased stabilization potency of elementary actin. Similar to the results reported previously (Tomanek et al., 2011), since the cytoskeleton is essential for the normal function of cells, the up-regulation of these genes could be a feedback to compensate for the reduction in cytoskeleton components caused by OA exposure.

It has been widely accepted that lysozymes play a crucial role in the oxygen-independent degradation of engulfed pathogen particles. However, only a few studies have investigated the impacts of OA on LZM of marine organisms, and controversial results have been reported in different organisms (Munari, 2014; Bresolin et al., 2016). For instance, a previous study conducted in *M. galloprovincialis* and *V. philippinarum* showed that OA exerted different impacts on the activities of LZM of these two bivalve species (Munari, 2014). The reason leading to the differences among investigations might be due to the fact that different types of lysozymes exist in different species (Takeshita et al., 2004; Xue et al., 2004; Zhao et al., 2007). For example, i-type and g-type LZMs, which may respond to OA differently, have been identified in *C. virginica* (Xue et al., 2004) and *C. farreri* (Zhao et al., 2007), respectively. In the present study, it has been shown that OA exposure led to a significant reduction in both the concentration and activity of LZM of hemocytes in the blood clam. These reductions indicated a hampered degradation of engulfed particles through the oxygen-independent pathway, which may also account for the reduction in phagocytosis under OA scenarios.

The increase in the production of *in vivo* NO induced by OA detected in the present study may be attributed to the following reasons. First, previous studies have suggested that reduced pH could promote the generation of NO in mammals or plants (Rõszer, 2012). Since it has been shown that OA exposure decreased the pH level of hemolymphs in blood clams (Zhao et al., 2017b), the acidosis caused by OA could be one of the reasons for the increased production of *in vivo* NO. Second, OA exposure could cause oxidative stress and lead to an increase in ROS, which may subsequently hamper the homeostasis of mitochondria and induce the generation of antioxidant enzymes, such as various reductases (Hu et al., 2015). On the one hand, since reductive NO can be produced from ${\mathrm{NO}}_{2}^{–}$ by reductases, the increase in reductases may promote the production of reductive NO (Rõszer, 2012). On the other hand, the generation of NO may be induced in response to ROS to stabilize mitochondria as well (Mao et al., 2013). Third, since *in vivo* oxidative NO is generated mainly through nitric oxide synthase (*NOS*), the detected up-regulation of the *NOS2* gene may also increase the production of oxidative NO under OA scenarios. Since the transcription of *NOS2* can be triggered by specific stimuli such as inflammation, the up-regulation of *NOS2* may be due to increased inflammatory reactions induced by OA stress (Ganster et al., 2001). Also, since the *in vivo* NO plays versatile roles in innate immunity, various consequences in terms of phagocytosis may be caused by the increased production of NO. On the one hand, as an active antimicrobial molecule, the increase in *in vivo* NO after OA exposure seems to indicate an enhanced ability to degrade engulfed particles. On the other hand, as one of the most important immune regulators, the induced production of *in vivo* NO could suppress immune responses such as phagocytosis (Mao et al., 2013).

In addition, according to previous studies, the hampered recognition of pathogen particles by hemocytes through PRRs (Liu et al., 2016; Castillo et al., 2017) and the re-allocation of energy among various life processes (Kurihara, 2008; Pan et al., 2015) could account for the reduction in phagocytosis detected as well. As been suggested in previous studies (Lannig et al., 2010; Pan et al., 2015; Liu et al., 2016), under the stress of OA, marine organisms may allocate more energy to critical life processes such as basal metabolism and internal ion regulation and therefore put a constraint on the energy available needed for the process of phagocytosis.

## Conclusion

The results obtained in the present study suggested that OA suppresses the process of phagocytosis through hampering the recognition of pathogen particles, inhibiting the process of engulfment by reducing the abundant of cytoskeleton components, as well as altering the oxygen-dependent and -independent degradation by enhancing the production of NO whereas reducing the concentration and activity of lysozyme.

## Author Contributions

WS and GL: conceived and designed the experimental plan; analyzed the data and drafted the manuscript. WS, JR, SZ, MY, and JF: performed the experiments.

## Conflict of Interest Statement

The authors declare that the research was conducted in the absence of any commercial or financial relationships that could be construed as a potential conflict of interest.
